# Cordycepin Prevents Bone Loss through Inhibiting Osteoclastogenesis by Scavenging ROS Generation

**DOI:** 10.3390/nu8040231

**Published:** 2016-04-20

**Authors:** Ce Dou, Zhen Cao, Ning Ding, Tianyong Hou, Fei Luo, Fei Kang, Xiaochao Yang, Hong Jiang, Zhao Xie, Min Hu, Jianzhong Xu, Shiwu Dong

**Affiliations:** 1 Department of Orthopedics, Southwest Hospital, Third Military Medical University, Chongqing 400038, China; lance.douce@gmail.com (C.D.); tianyonghou@126.com (T.H.); luofly1009@21cn.com (F.L.); xiezhao54981@163.com (Z.X.); 2 Department of Biomedical Materials Science, School of Biomedical Engineering, Third Military Medical University, Gaotanyan Street No. 30, Chongqing 400038, China; zhenyacy@163.com (Z.C.); chrisdawn@163.com (N.D.); kangfeilove2007@126.com (F.K.); xcyang@tmmu.edu.cn (X.Y.); jh_tmmu@126.com (H.J.); 3 Research Center for Molecular Medicine, Kunming University, Kunming 650000, China; humin999@foxmail.com

**Keywords:** cordycepin, osteoclasts, ROS, IRF-8, osteoporosis

## Abstract

Cordycepin was previously reported to have anti-tumor, anti-inflammatory and anti-oxidant activity. However, the potential role of cordycepin in bone metabolism and cell biology of osteoclasts remains unclear. In our study, we focused on the *in vitro* effects of cordycepin on osteoclastogenesis and its *in vivo* effects in ovariectomized (OVX) mice. Osteoclast differentiation, formation and fusion were evaluated by Tartrate-resistant acid phosphatase (TRAP) stain, focal adhesion stain and fusion assay, respectively. Osteoclastic bone resorption was evaluated by pit formation assay. Reactive oxygen species (ROS) generation and removal were detected by the ROS assay. OVX mice were orally administered with 10 mg/kg of cordycepin daily for four weeks. *In vitro* results revealed that cordycepin inhibited receptor activator of nuclear factor κB ligand (RANKL)-induced osteoclast differentiation, formation, fusion and bone resorption activity. We further proved that cordycepin treatments scavenged the generation of ROS, upregulated interferon regulatory factor 8 (IRF-8) and suppressed the activity of nuclear factor of activated T cells c1 (NFATc1) during osteoclastogenesis. *In vivo* results indicated cordycepin prevents bone loss, rescues bone microarchitecture, and restores bone mineralization in OVX mice. Our observations strongly suggested that cordycepin is an efficient osteoclast inhibitor and hold potential therapeutic value in preventing bone loss among postmenopausal osteoporosis patients.

## 1. Introduction

Bone homeostasis is maintained by a continuous and balanced bone-resorbing and bone-forming process. In this process, the two most crucial cell types are osteoclast (OC) and osteoblast (OB). Osteoblasts derived from mesenchymal stem cells (MSCs) are in charge of bone-forming process including bone matrix formation and mineralization. Osteoclasts, on the other hand, are derived from hematopoietic stem cells (HSCs) or monocytes/macrophage progenitor cells responsible for bone resorption [1]. The two cell types collaborate together balancing and orchestrating the process of bone remodeling. Disturbance of the balance may lead to impairment of bone metabolism causing diseases such as osteoporosis and osteopetrosis [2]. Osteoporosis has become a public health issue with the increasing rate of hip fracture, the most serious outcome of osteoporosis [3]. The bone loss of osteoporosis results from increased bone resorption amount over bone formation. On the cellular level, it can be elucidated by over-activation of osteoclasts and/or inhibition of osteoblasts [4,5].

Mature osteoclasts are bone-specific polykaryons. Two of the most important regulating factors during osteoclasts differentiation are receptor activator of nuclear factor κB ligand (RANKL) and macrophage-colony stimulating factor (M-CSF) [6]. Binding of RANKL to its receptor results in the initiation of the TNF receptor-associated factor (TRAF) 6 signaling, which activates nuclear factor-κB (NF-κB), Akt, and mitogen-activated protein (MAP) kinase and eventually activates nuclear factor of activated T cells c1 (NFATc1), the master regulator in osteoclastogenesis, [7,8] leading to osteoclast proliferation, differentiation, and maturation [2,9]. Interferon regulatory factor-8 (IRF-8) is a suppressive transcription factor that inhibits osteoclastogenesis by inhibiting the function and expression of NFATc1 [10]. During RANKL induced osteoclast formation, reactive oxygen species (ROS) has also been shown playing important roles in the process of differentiation, survival, activation and bone resorption [11,12,13,14]. MAP kinases including c-Jun N-terminal kinase (JNK) and p38 MAP kinase (p38) can be activated by ROS [13,15]. ROS over-generation is usually associated with pathological conditions caused by bone resorption such as inflammatory arthritis and estrogen-deficient osteoporosis [16,17,18]. Postmenopausal osteoporosis is a disease marked with excessive bone resorption that is caused by estrogen deficiency. Estrogen is crucial in maintaining bone health due to its antioxidant activity in osteoclasts (OCs) and stimulating effects in osteoblasts (OBs). Although the details of the mechanisms are unclear, excess production of ROS due to estrogen deficiency is one of the main pathogenic factors in postmenopausal osteoporosis [17,19]. A recent study also has drawn attention to ROS as a potential target in treating osteoporosis [20].

*Cordyceps sinensis* is a caterpillar fungus that has been used in Chinese traditional medicine for more than 1700 years since Jin Dynasty. The major functional bioactive component isolated from cordyceps is called cordycepin (3′-deoxyadenosine). Cordycepin is a natural derivative of the nucleoside adenosine with only one absence of oxygen in the 3′ position of the ribose part [21,22,23]. The biological function of cordycepin has been widely explored for its anti-tumor, anti-inflammatory and anti-oxidant activity in various disease models [24,25,26,27,28,29]. Previous studies implied that cordycepin was closely associated with ROS-related cell biology. However, little is known about the effects of cordycepin in osteoclasts and in the skeletal system.

In the current study, we demonstrated the inhibitory effects of cordycepin in osteoclastogenesis and preventative effects on bone loss in OVX mice. Mechanistically, cordycepin suppressed osteoclastogenesis through scavenging ROS generation and activating (IRF-8).

## 2. Experimental Section

### 2.1. Reagents

RAW264.7 cells were obtained from the American Type Culture Collection (Rockville, MD, USA). Recombinant Mouse RANKL and Recombinant Mouse M-CSF were purchased from R&D Systems (Minneapolis, MN, USA). Antibodies against p38, p-p38, FoxO1, Nrf2, NFATc1, IRF-8 and β-actin were purchased from Santa Cruz Biotechnology (Santa Cruz, CA, USA). Antibody against DC-STAMP was purchased from Millipore (Darmstadt, Germany). Antibody against ATP6v0d2 was purchased from ABcam (Cambridge, UK). Osteo Assay Surface for bone resorption was purchased from Corning (Corning, NY, USA). TRAP stain kit was obtained from Sigma-Aldrich (St. Louis, MO, USA). Actin Cytoskeleton and Focal Adhesion Staining Kit was purchased from Millipore (Darmstadt, Germany). Membrane dye DiI and Cell Tracker Green were obtained from Life Technologies (Carlsbad, CA, USA). Alpha minimal essential Medium (α-MEM) and fetal bovine serum (FBS) was purchased from Gibco (life technologies, Carlsbad, CA, USA). Penicillin-streptomycin solution was obtained from Hyclone (Thermo Scientific, Waltham, MA, USA). Cordycepin was purchased from Sigma-Aldrich (St. Louis, MO, USA).

### 2.2. Mice and Treatments

Eight-week-old female C57BL/6 mice were provided by the animal center of Third Military Medical University. All experimental procedures were approved by Third Military Medical University and performed according to guidelines of laboratory animal care and use. All efforts were made to reduce the number of animals tested and their suffering. Mice were divided into three groups: sham operated mice (sham, *n* = 3), OVX mice (control, *n* = 3) and cordycepin treated OVX mice (Cordycepin, *n* = 3). Mice were weighed daily and concentration was calculated for the dose of 10 mg/kg (body weight) bw for cordycepin administration. Cordycepin was dissolved in distilled water and administrated orally daily using a feeding needle for 28 days. Control groups received only water. All treated mice were sacrificed by cervical dislocation one day after last administration.

### 2.3. CCK-8 Cell Proliferation and Viability Assay

RAW264.7 cells and primary bone marrow macrophages (BMMs) were seeded (2 × 10^3^ per well) into 96-well plates and were cultured overnight. Cells were induced with M-CSF (50 ng/mL) and RANKL (50 ng/mL) for 24 h or 72 h with different treatment dosages of cordycepin. Cell proliferation and viability were evaluated by Cell Counting Kit-8 (CCK8, Dojindo, Japan) reagent at 24 h, and 72 h according to the manufacturers’ instructions. The absorbency of cells was measured using a 96-well plate reader at 450 nm.

### 2.4. Flow Cytometry Cell Apoptosis Assay

Primary BMMs apoptosis rate was detected by FCM with Annexin V-FITC Apoptosis Detection Kit (KeyGEN) following to the manufacturer’s instructions. One hundred milliliters of 10^5^ cells in suspension were stained with kit solution (Annexin-V-FITC and PI) in dark for 15 min. The apoptosis rate was assayed by using FACSCalibur Flow Cytometry (BD, Triangle, NC, USA) at 488 nm.

### 2.5. Reactive Oxygen Species (ROS) Assay

Primary BMMs (5 × 10^3^ cells/well in 96 well plates) were cultured in DMEM medium (10% FBS, 1% antibiotics) containing M-CSF (50 ng/mL) and RANKL (50 ng/mL) for 24 h and each well was replaced with DMEM medium (10% FBS, 1% antibiotics). Cells were then treated with different concentrations of cordycepin and ROS positive control group was set. Intracellular ROS level was measured by 2′, 7′-dichlorofluorescein diacetate (DCFH), which can be oxidized into fluorescent DCF. After fixing, the cells were washed in 1× PBS and then incubated in the dark for 30 min with 10 μM DCFH-DA. Cells were counterstained with DAPI for better view of total cell number. Images were taken using the fluorescence of DCF by fluorescence microscopy.

### 2.6. Tartrate-Resistant Acid Phosphatase (TRAP) Staining

Primary BMMs were cultured in α-minimal essential medium (MEM) containing 10% FBS and 1% Penicillin-streptomycin solution with M-CSF (50 ng/mL) and RANKL (50 ng/mL) for 72 h. For TRAP stain, cells were cultured in a 96-well plate at a density of 5 × 10^3^ cells/well. Cells were fixed in 4% paraformaldehyde for 20 min after 72-h induction and then stained with TRAP staining solution (0.1 mg/mL of naphthol AS-MX phosphate, 0.3 mg/mL of Fast Red Violet LB stain) according to the manufacturer. TRAP-positive multinucleated cells containing nuclei of one, 2–3 or more than 3 were counted.

### 2.7. Pit Formation Assay

Primary BMMs were incubated in 96-well plates (Corning Osteo Assay Surface), 2 × 10^3^ cells/well. Primary BMMs were incubated in 48-well plates covered with bovine bone slices, 1 × 10^4^ cells/well. Cells were induced with RANKL (50 ng/mL) and M-CSF (50 ng/mL) for 96 h with different concentration of cordycepin treatments. Methylene blue stain was performed to evaluate the resorption area on bone slices. Bleach solution was added to 96-well osteo surface plates to remove cells. Detailed analysis of pit formation area was described in our previous study [30].

### 2.8. Actin Cytoskeleton and Focal Adhesion Staining

Primary BMMs were incubated in 96-well plate (5 × 10^3^ cells/well) and induced for 72 h with cordycepin treatments. Procedures were described in previous study [30]. In brief, on Day 4, cells were washed and fixed for permeabilization. After blocking, primary antibody (Anti-Vinculin) was then diluted to a working concentration (1:300) in blocking solution, and cells were incubated for 1 h at room temperature. Secondary antibody (Alexa Fluor 488 Goat Anti-Mouse IgG (H + L) Antibody, Invitrogen) (1:500) and TRITC conjugated Phalloidin (1:500) was diluted in 1× PBS and cells were incubated for 1 h at room temperature. Nuclei counterstaining was performed by DAPI (1:1000) for 5 min followed by fluorescence microscopy observation.

### 2.9. Fusion Assay

For evaluation of the effects of cordycepin on osteoclasts fusion, fussion assay was adopted as described in previous study [31]. Primary BMMs were induced with RANKL (50 ng/mL) and M-CSF (50 ng/mL) for 72 h in 6-well plates. Then, cells were labeled with either membrane dye DiI or cell content marker Cell tracker green. After incubation for 30 min at room temperature, cells labeled with DiI were scraped and put onto the well containing cells labeled with cell tracker green. The co-plated cells were then incubated together for 2 h before removal of the medium. Fluorescence microscopy was adopted for observation. Image J software (Java open resource) was adopted for the analysis of membrane merge rate.

### 2.10. Real-Time qPCR

Total RNA was isolated using Trizol reagent (Life Technologies). Single-stranded cDNA was prepared from 1 μg of total RNA using reverse transcriptase with oligo-dT primer according the manufacturer’s instructions (Promega, Fitchburg, WI, USA). Two microliters of each cDNA was subjected to PCR amplification using specific primers for FoxO1, Nrf2, ATP6v0d2, NFATc1, mitf, OSCAR, DC-STAMP, CD9 and IRF-8 with detailed information in Table 1.

### 2.11. Immunoblotting

Cells were lysed in a lysis buffer containing 10 mM Tris, pH 7.2, 150 mM NaCl, 5 mM EDTA, 0.1% SDS, 1% Triton X-100, and 1% deoxycholic acid. For Western blots, 30 μg of protein samples were subjected to SDS-PAGE followed by transfer onto PVDF membranes. After blocking in 5% skim milk, membranes were incubated with rabbit antibodies against Akt (1:1000, Santa Cruz), p-Akt (1:500; Santa Cruz), p38 (1:500, Santa Cruz), p-p38 (1:500, Santa Cruz), FoxO1 (1:500, Santa Cruz), Nrf2 (1:500, Santa Cruz), NFATc1 (1:500, Santa Cruz), IRF-8 (1:500, Santa Cruz), DC-STAMP (1:1000, Millipore) and β-actin (1:500, Santa Cruz) overnight at 4 °C followed by 1 h-incubation with secondary antibody (1:2000). Blots against β-actin served as loading control.

### 2.12. μCT Analysis and Histological Analysis

For μCT analysis, Bruker MicroCTSkyscan 1272 system (Kontich, Belgium) with an isotropic voxel size of 10.0 μm was used to image the whole femur. Scans were conducted in 4% paraformaldehyde and used an X-ray tube potential of 60 kV, an X-ray intensity of 166 μA, and an exposure time of 1700 ms. For trabecular bone analysis of the distal femur, an upper 3-mm region beginning 0.8 mm proximal to the most proximal central epiphysis of the femur was contoured. For cortical bone analysis of femur (2D analysis), a 0.5-mm region beginning 4.5 mm proximal to the most proximal central epiphysis of the femur. Trabecular bones were threshholded at 86–255 (8 bit grey scale bitmap). μCT scans of whole body of mice (except skull) were performed using isotropic voxel sizes of 148 μm. Reconstruction was accomplished by Nrecon (Version 1.6.10; Kontich, Belgium). 3D images were obtained from contoured 2D images by methods based on distance transformation of the grey scale original images (Version 3.0.0; CTvox, Kontich, Belgium). 3D and 2D analysis were performed using software CT Analyser (Version 1.15.4.0; Kontich, Belgium). All images presented are representative of the respective groups.

For the bone histological analysis, femurs were dissected and fixed in 4% paraformaldehyde in PBS for 48 h. Femurs were then decalcified by daily change of 15% tetrasodium EDTA for 2 weeks. Tissues were dehydrated by passage through an ethanol series, cleared twice in xylene, embedded in paraffin, and sectioned at 8 μm thickness along the coronal plate from anterior to posterior. Decalcified femoral sections were stained with hematoxylin and eosin (H&E) and Masson.

### 2.13. Statistics

All data are representative of at least three experiments of similar results performed in triplicate unless otherwise indicated. Data are expressed as mean ± SD. One-way ANOVA followed by Student–Newman–Keuls *post hoc* tests was used to determine the significance of difference between results, with *p* < 0.05 being regarded as significant.

## 3. Results

### 3.1. Toxicity Evaluation of Cordycepin on RANKL-Induced Osteoclastogenesis

**Figure 1:**
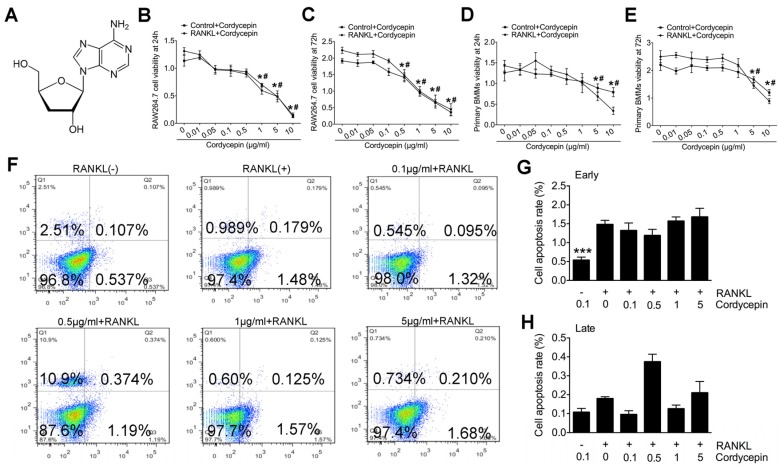
Toxicity evaluation of cordycepin on receptor activator of nuclear factor κB ligand (RANKL)-induced osteoclastogenesis: (**A**) Chemical formula of cordycepin; (**B,C**) Cell counting kit-8 (CCK8) analysis of cell viability of RAW264.7 cells treated with RANKL (50 ng/mL), macrophage-colony stimulating factor (M-CSF) (50 ng/mL) and different dosages of cordycepin for 24 h (**B**) and 72 h (**C**) respectively; (**D**,**E**) CCK8 analysis of cell viability of primary bone marrow macrophages (BMMs) treated with RANKL (50 ng/mL), M-CSF (50 ng/mL) and different dosages of cordycepin for 24 h (**E**) and 72 h (**D**) respectively; (**F**) FCM analysis of cell apoptosis rate of primary BMMs treated with RANKL (50 ng/mL) and M-CSF (50 ng/mL) for 24 h with different dosages of cordycepin treatments; (**G**) analysis of early stage cell apoptosis rate; and (**H**) analysis of late stage cell apoptosis rate. Data in the figures represent average ± SD. (* among Control + Cordycepin groups, # among RANKL + Cordycepins groups) * *p*< 0.05, *** *p*< 0.001, # *p*< 0.05 based on one way analysis of variance (ANOVA).

The chemical formula of cordycepin is shown (Figure 1A). Toxicity of cordycepin was evaluated by CCK-8 assay and flow cytometry (FCM) cell apoptosis assay. RAW264.7 cells and primary bone marrow monocytes (BMMs) were treated with RANKL (50 ng/mL) and M-CSF (50 ng/mL) for 24 h and 72 h respectively with different dosages of cordycepin (Figure 1). The CCK-8 results showed that cordycepin was toxic in RAW264.7 cells at the concentration of 1 μg/mL (24 h) and 0.5 μg/mL (72 h) (Figure 1B,C), it also revealed that cordycepin started to affect cell viability of BMMs at the concentration of 5 μg/mL (Figure 1D,E). FCM was then performed to evaluate the effects of cordycepin on cell apoptosis rate during osteoclastogenesis in primary BMMs (Figure 1F). The results showed that cells treated with cordycepin at a concentration of 5 μg/mL presented a slightly higher early apoptosis rate yet with no significance (Figure 1G). The results were similar for the late apoptosis rate (Figure 1H). In combination with the FCM results, the decease of cell viability detected by CCK-8 does not indicate toxicity but suppression of cell growth.

### 3.2. Cordycepin Attenuates TRAP-Positive Osteoclast Number in a Dose-Dependent Way

**Figure 2:**
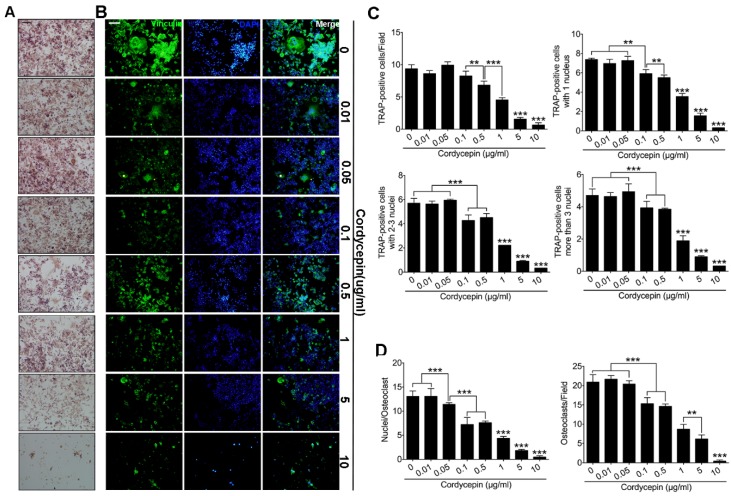
Cordycepin inhibits RANKL-induced osteoclast formation in a dose-dependent way. (**A**) Representative images of primary BMMs stained for Tartrate-Resistant Acid Phosphatase (TRAP) (red) treated with RANKL (50 ng/mL) and M-CSF (50 ng/mL) for 72 h. Eight different groups were set according to different concentrations of cordycepin treatment (0 μg/mL, 0.01 μg/mL, 0.05 μg/mL, 0.1 μg/mL, 0.5 μg/mL, 1 μg/mL, 5 μg/mL, and 10 μg/mL). Scale bar represents 200 μm. (**B**) Representative images of focal and adhesion staining of primary BMMs treated with RANKL (50 ng/mL) and M-CSF (50 ng/mL) for 72 h. Double immunofluorescence for nuclei using DAPI (blue), F-actin using TRITC-conjugated Phalloidin (red) and vinculin using Vinculin Monoclonal Antibody (green) were shown. Scale bar represents 40 μm. (**C**) Number of TRAP (+) cells, TRAP (+) cells with one nucleus, TRAP (+) cells with 2–3 nuclei and TRAP (+) cells with more than 3 nuclei in each well (96-well plate). (**D**) Quantification of osteoclasts (nuclei ≥ 3) in each field and average osteoclasts nuclei number in each field. Data in the figures represent average ± SD. ** *p* < 0.01, *** *p* < 0.001 based on one way ANOVA.

To evaluate the effects of cordycepin on RANKL-induced osteoclast formation, primary BMMs were treated with RANKL (50 ng/mL) and M-CSF (50 ng/mL) for three days with different concentrations of cordycepin. Groups were set according to the different treating concentrations of cordycepin (0 μg/mL, 0.01 μg/mL, 0.05 μg/mL, 0.1 μg/mL, 0.5 μg/mL, 1 μg/mL, 5 μg/mL, and 10 μg/mL). TRAP stain was performed to evaluate the effects of cordycepin on osteoclastogenesis (Figure 2A). TRAP positive cells in each well (96-well plate) were counted and sorted according to the nuclei number. The results demonstrated that the inhibitory effects of cordycepin started at the concentration of 0.1 μg/mL. With cordycepin treatment above the dosage of 1 μg/mL, the inhibitory effects become extremely obvious. According to the nuclei number in TRAP-positive cells, we discovered that cells with 1 nucleus, 2–3 nuclei and more than 3 nuclei showed similar trends (Figure 2C). We concluded that cordycepin has an inhibitory effect on RANKL-induced osteoclastogenesis in a dose-dependent way.

### 3.3. Cordycepin Inhibits RANKL-Induced Osteoclast Fusion in a Dose-Dependent Way

**Figure 3:**
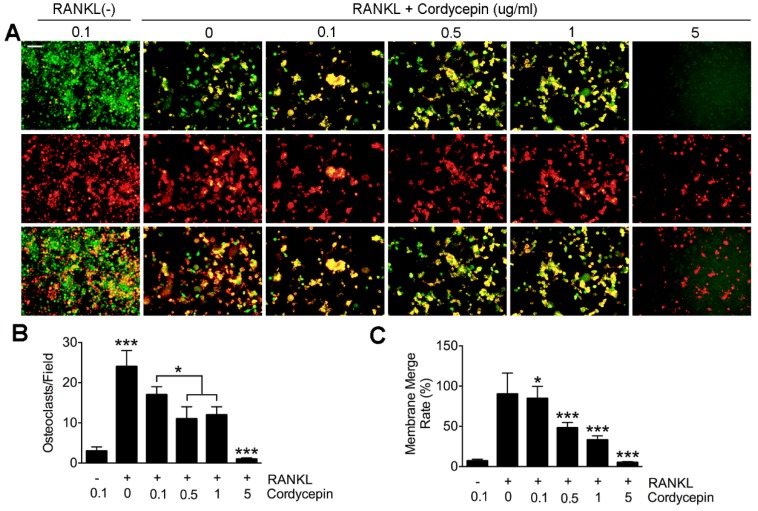
Cordycepin inhibits RANKL-induced osteoclast fusion in a dose-dependent way. Cells were labeled with either membrane dye DiI (red color) or cell content marker Cell tracker (green color) and then mixed together for fusing process. (**A**) Representative images of fusion assay. Primary BMMs treated without RANKL, M-CSF and cordycepin were set as blank control group. Five different groups were set according to different concentrations of cordycepin treatment (0 μg/mL, 0.1 μg/mL, 0.5 μg/mL, 1 μg/mL, and 5 μg/mL). (**B**) Quantification of osteoclasts (nuclei ≥ 3) in each field. (**C**) Membrane merge rate of osteoclasts in each field. Scale bar represents 40 μm. Data in the figures represent average ± SD. * *p* < 0.05, *** *p* < 0.001 based on one way ANOVA.

To further investigate the effects of cordycepin on osteoclast fusion, we performed actin cytoskeleton and focal adhesion (FAK) stain and fusion assay (Figure 2 and Figure 3). The results we obtained were generally consistent with the results of TRAP stain. Primary BMMs were treated with RANKL (50 ng/mL) and M-CSF (50 ng/mL) for three days with different concentrations of cordycepin. Group settings were the same with TRAP stain. As for fusion assay, groups were set as RANKL (−), RANKL (+) with no cordycepin and RANKL with cordycepin (0.1 μg/mL, 0.5 μg/mL, 1 μg/mL, and 5 μg/mL). From the FAK results, we found that osteoclast number decreased significantly when the concentration reached 0.1 μg/mL. However, the average nuclei number of osteoclasts started to show significant decrease at the dosage of 0.05 μg/mL (Figure 2D). In the fusion assay, the osteoclast number was decreased by cordycepin treatments as expected. It was worthy to notice that the fusion process was inhibited more intensively by cordycepin (Figure 3B,C). After analysis, the results showed that membrane merge rate was down regulated by cordycepin from the dosage of 0.1 μg/mL (Figure 3C). We concluded that cordycepin is inhibitory on the fusion of osteoclasts in a dose-dependent way.

### 3.4. Cordycepin Reduces Osteoclastic Bone Resorption Activity in a Dose-Dependent Way

**Figure 4:**
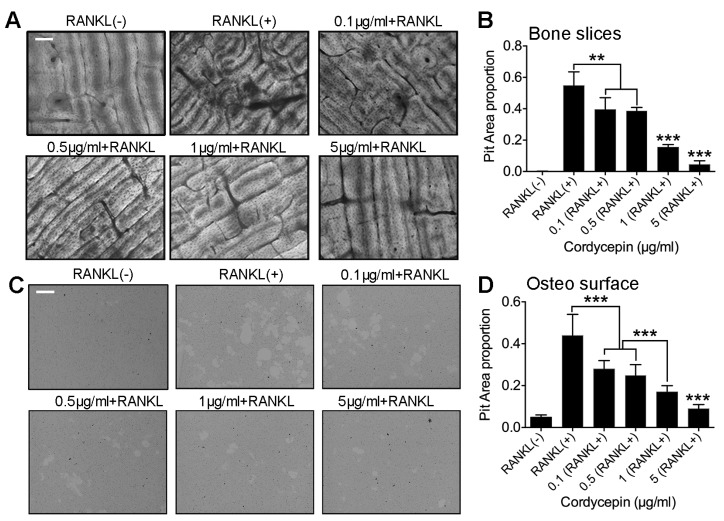
Cordycepin reduces osteoclastic bone resorption activity in a dose-dependent way. (**A**) Representative images of primary BMMs cultured on bovine slices treated with RANKL (50 ng/mL) and M-CSF (50 ng/mL) for five days with different concentrations of cordycepin treatment (0 μg/mL, 0.1 μg/mL, 0.5 μg/mL, 1 μg/mL, and 5 μg/mL). (**B**) Quantification of RANKL induced osteoclastic bone resorption in the bone slices group. (**C**) Representative images of Osteoassay surface 96-well plate after removal of osteoclasts. (**D**) Quantification of RANKL induced osteoclastic bone resorption in the osteo surface group. Scale bar represents 400 μm. Data in the figures represent average ± SD. ** *p* < 0.01, *** *p* < 0.001 based on one way ANOVA.

To further detect the effects of cordycepin on the osteoclastic bone resorption activity, we performed pit formation assay. Both bovine bone slices and Corning Osteo Assay Surface (96-well plates) were used for the experiment (Figure 4A,C). To our expectation, the results of the two methods were similar. Primary BMMs were treated with RANKL (50 ng/mL) and M-CSF (50 ng/mL) for five days with different concentrations of cordycepin on bone slices or Osteo Surface. From the statistical data, we found that groups treated with 0.1 μg/mL cordycepin started to show significant decrease in the bone resorption area compare to groups treated with only RANKL and M-CSF (Figure 4B,D). When the concentration of cordycepin reached to 5 μg/mL, the bone resorption activity almost disappeared (Figure 4B,D). We concluded that cordycepin could also attenuate the bone resorption activity of osteoclasts in a dose-dependent way.

### 3.5. Cordycepin Scavenges ROS Generation and Activates FoxO1 and Nrf2 Expression

**Figure 5:**
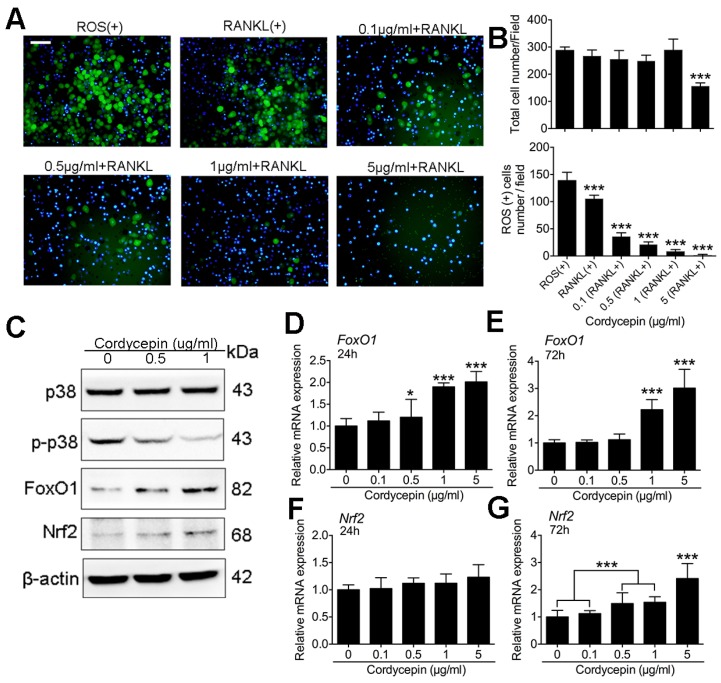
Cordycepin scavenges ROS generation and activates FoxO1 and Nrf2 expression. (**A**) Representative images of ROS positive BMMs during RANKL induced osteoclastogenesis treated with different concentrations of cordycepin (0 μg/mL, 0.1 μg/mL, 0.5 μg/mL, 1 μg/mL, and 5 μg/mL). (**B**) Quantification of total cell number and ROS positive cell number in each field. (**C**) Representative Western blot images of p38, p-p38, FoxO1, Nrf2 and β-actin. For blotting of p38 and p-p38, cells were pretreated with different dosages of cordycepin for 24 h and then induced with RANKL and M-CSF for 15 min. For FoxO1 and Nrf2, cells were treated with different dosages of cordycepin for 72 h with RANKL and M-CSF. (**D**,**E**) Relative mRNA expression levels of *FoxO1* at 24 h (**D**) and 72 h (**E**) during osteoclastogenesis. (**F**,**G**) Relative mRNA expression levels of *Nrf2* at 24 h (**F**) and 72 h (**G**) during osteoclastogenesis. Scale bar represents 200 μm. Data in the figures represent average ± SD. * *p* < 0.05, *** *p* < 0.001 based on one way ANOVA.

To reveal the underlying mechanism of the inhibitory effects of cordycepin on RANKL-induced osteoclastogenesis, we measured the ROS production by DCFH treated with different dosages of cordycepin during osteoclastogenesis (Figure 5A). The results showed that RANKL treatment alone without cordycepin could significantly increase the generation of ROS. Meantime, treatment of cordycepin remarkably decreased the ROS level increased by RANKL. The scavenging effects of cordycepin on ROS production is also dose-dependent, concentration of cordycepin at 5 μg/mL had the best scavenging activity (Figure 5B). To further explore the molecular mechanism, Western blot was performed on the expression of p38, p-p38, FoxO1 and Nrf2. The results showed that cordycepin treatment down regulated the phosphorylation of p38 (Figure 5C). In addition, expression of FoxO1 and Nrf2 were both increased by cordycepin treatments. The qPCR results demonstrated similar results showing that the mRNA expression of FoxO1 and Nrf2 were increased by cordycepin treatment in a dose-dependent way (Figure 5D–G).

### 3.6. Cordycepin Activates IRF-8 and Suppresses NFATc1 Expression during Osteoclastogenesis

**Figure 6:**
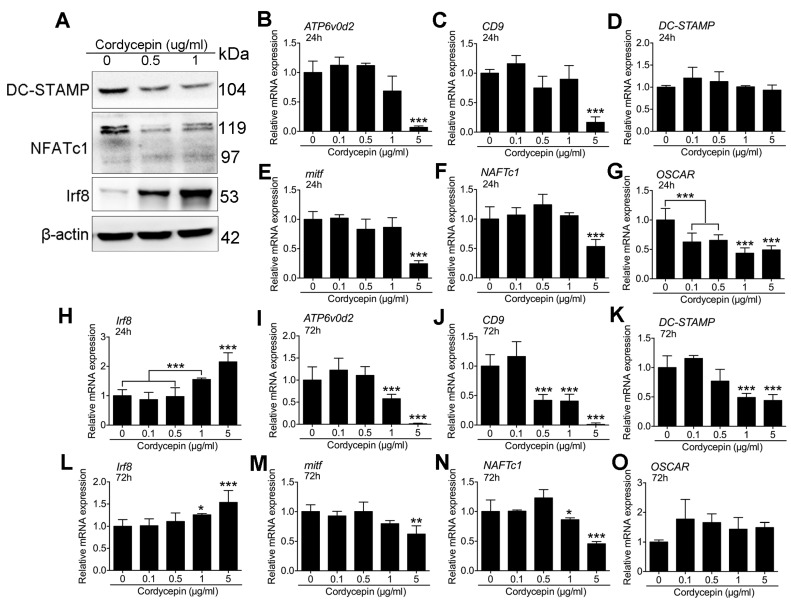
Cordycepin activates IRF-8 and suppresses NFATc1 expression during osteoclastogenesis. (**A**) Representative Western blot images of, DC-STAMP, NFATc1, IRF-8 and β-actin. Cells were treated with different dosages of cordycepin for 72 h with RANKL and M-CSF before protein extraction. (**B**–**H**) Relative mRNA expression levels of *ATP6v0d2* (**B**); *CD9* (**C**); *DC-STAMP* (**D**); *mitf* (**E**); *NFATc1* (**F**); *OSCAR* (**G**); and *IRF-8* (**H**) at 24 h during osteoclastogenesis. (**I**–**O**) Relative mRNA expression levels of *ATP6v0d2* (**i**); *CD9* (**J**); *DC-STAMP* (**K**); *mitf* (**L**); *NFATc1* (**M**); *OSCAR* (**N**); and *IRF-8* (**O**) at 72 h during osteoclastogenesis. Data in the figures represent average ± SD. * *p* < 0.05, ** *p* < 0.01, *** *p* < 0.001 based on one way ANOVA.

To further explore the molecular mechanism of the inhibitory effects of cordycepin on RANKL-induced osteoclastogenesis. We performed Western blot on several fusion related molecules DC-STAMP. We also tested the expression of the master regulatory gene, NFATc1 (Figure 6A). The results indicated that expression of DC-STAMP was down regulated by cordycepin treatments (Figure 6A). On the other hand, we tested the expression of IRF-8, a negative regulatory gene. The results showed that IRF-8 was upregulated by cordycepin treatments (Figure 6A). We also performed qPCR on *ATP6v0d2*, *DC-STAMP*, *NFATc1* and *IRF-8*, most of the results were consistent with the WB results (Figure 6B,F,H,I,K,L,N). The mRNA expression of DC-STAMP showed no obvious change at 24 h, but started to decrease at 72 h (Figure 6D,K). Besides, we also performed qPCR on the expression of other osteoclast related genes like *OSCAR*, *mitf* and *CD9*. To our expectation, the genes expressions were changed by the treatments of cordycepin. *ATP6v0d2* and *CD9* were significantly down regulated at 72 h and *OSCAR* was down regulated at 24 h while *mitf* inhibition showed no relevance with time (Figure 6C,E,G,J,M,O).

### 3.7. Cordycepin Prevents Bone Loss in OVX Mice

**Figure 7:**
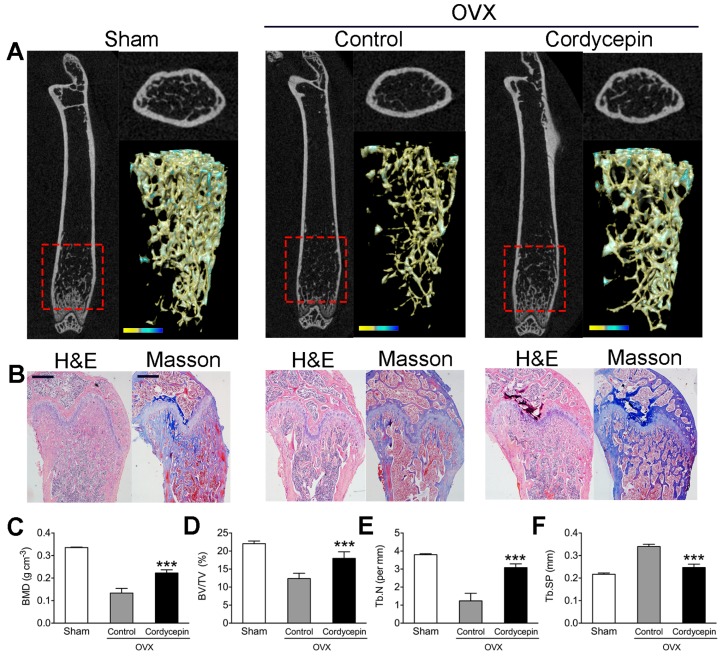
Cordycepin preventes bone loss in ovariectomized (OVX) mice: (**A**) Representative microCT longitudinal section images of femurs, cross-sectional view of the distal femurs and reconstructed trabecular structure of the region of interest (red dashed box). Color scale bar represents bone mineral density level. (**B**) Representative images of histological slides of H&E stain and Masson stain focusing on the metaphyseal region of the distal femur from mice of different groups. Scale bar represents 800 μm. (**C**–**F**) Quantitative microCT analysis of average femur length, distal femoral volumetric bone mineral density (BMD), trabecular bone volume fraction (BV/TV), trabecular number (Tb. N) and trabecular separation (Tb. Sp) in each group. Data in the figures represent average ± SD (*n* = 3). *** *p* < 0.001 (*vs.* Control) based on one way ANOVA.

To determine the *in vivo* anti-bone resorption effects of cordycepin, we adopted OVX mice as an osteoporosis model with excessive osteoclastic bone loss. Mice were weighed daily and concentrations were calculated for the doses of 10 mg/kg bw. All treated mice were sacrificed by cervical dislocation one day after the four-week orally administration. The results showed that cordycepin administration could significantly increase of the distal femoral volumetric bone mineral density (BMD), trabecular bone volume fraction (BV/TV), trabecular number (Tb. N) and decrease of trabecular separation (Tb. Sp) of OVX mice (Figure 7A). H&E stain results were consistent with the microCT results and Masson stain suggested an increase of new bone formation in mice administered with cordycepin (Figure 7B). Quantification of our observations indicated that cordycepin could reverse the bone loss of osteoporotic mice (Figure 7C).

## 4. Discussion

ROS plays a dual role in bone homeostasis. In physiological conditions, ROS is necessary for the differentiation and survival of osteoclast maintaining its normal function in bone remodeling [12]. In pathological conditions such as inflammation and bone fracture, over production of ROS is always linked with excessive bone loss [32,33]. Postmenopausal osteoporosis is a disease marked with over bone resorption caused by estrogen deficiency. Estrogen is crucial in maintaining bone health with its antioxidant activity against OCs and stimulating effects in osteoblasts. Although the detailed mechanism remains unclear, over production of ROS due to estrogen deficiency is one of the main pathogenesis in postmenopausal osteoporosis [17,19]. The role of ROS during osteoclastogenesis is essential. MAP kinases including JNK and p38 are crucial for osteoclast differentiation and both of these two genes can be activated by ROS [13,15]. Recently, it has also been proven that ROS production in OC and intracellular hydrogen peroxide accumulation is critical for osteoclastogenesis and skeletal homeostasis [34]. To prevent the oxidative stress damage caused by over accumulation of ROS, several genes including FoxOs and Nrf2 will be activated to transcribe antioxidant enzymes [35,36]. In our study, we found that FoxO1 was activated with treatments of cordycepins. Oxidative stress could activate FoxOs through JNK pathway [37,38], scavenging ROS generation with cordycepin should reduce the expression of FoxOs theoretically. However, we discovered that FoxO1 were up regulated on the mRNA level and also protein level. We also found that Nrf2 was up regulated by the treatments of cordycepin. In recent studies, Nrf2 was reported attenuating osteoclasts differentiation through intracellular ROS signaling [39,40]. It is also worthy to notice that p38 activation was inhibited by treatments of cordycepin indicating a potential apoptosis inducing effects [41]. This result is consistent with the hypothesis of several studies on the apoptosis-inductive effects of cordycepin [42,43]. We concluded that enhanced FoxO1 and Nrf2 expressions in turn accelerate the scavenging of ROS generation during osteoclastogenesis. However, we cannot yet distinguish if ROS was scavenged directly by cordycepin or indirectly by activation of FoxO1 and Nrf2.

Numerous antioxidant compounds have been proved inhibitory in osteoclastogenesis in the past studies [44,45,46]. Cordycepin was also reported being protective towards specific cell types by reducing oxidative stress. At the same time, reports showed that the anti-tumor effect of cordycepin was realized through ROS induced apoptosis [42,47]. In our study, we observed the remarkable antioxidant effects of cordycepin during RANKL-induced osteoclastogenesis. Taking consideration of the anti-inflammation effects, pro-inflammatory cytokines and NF-κB signaling pathways inhibition activity of cordycepin [48,49], it is reasonable that cordycepin can also be suppressive on osteoclastogenesis. Instead of the NF-κB signaling pathways, we focused on ROS related signaling network in our study. It is still worthy to test if the inhibitory effects of cordycepin on NF-κB signaling exist during osteoclastogenesis. It will also be interesting to study on the role of cordycepin in ROS-induced apoptosis in the late stages during osteoclast maturation.

IRF-8 is a member of the IRF family specifically expressed in immune cells and has been shown as regulatory on myeloid cell development [50]. IRF-8 was discovered to be a critical inhibitory regulator during osteoclastogenesis to maintain bone health [10]. The suppressive effect of IRF-8 in osteoclastogenesis is realized through inhibition of NFATc1. In our study, we found that IRF-8 expression was enhanced by cordycepin treatments and NFATc1 expression was decreased in accordance. The results showed that cordycepin could also inhibit osteoclastogenesis through activating IRF-8 and thus down regulating NFATc1. Recently, a report stated that IRF-8 is inhibitory on inflammation, oxidative stress and neuronal apoptosis [51]. It is interesting because we also tested the alteration of IRF-8 under a ROS dominant signaling circumstance.

In conclusion, we demonstrated that cordycepin inhibits RANKL-induced osteoclastogenesis mostly by scavenging ROS generation. In particular, cordycepin up regulated FoxO1 together with Nrf2 accelerated the elimination of ROS. We also found that IRF-8 was up regulated by cordycepin treatments leading to further inhibition of osteoclastogenesis by suppressing NFATc1. Moreover, administration of cordycepin could significantly prevent bone loss in OVX mice. Our results suggested that dietary or supplementary cordycepin intake might be of potential therapeutic value for the prevention of postmenopausal osteoporosis.

## Figures and Tables

**Table 1:** Primer sequences for PCR.

Primers	Forward	Reverse	Tm (°C)
DC-STAMP	5′-TTATGTGTTTCCACGAAGCCCTA-3′	5′-ACAGAAGAGAGCAGGGCAACG-3′	62
CD9	5′-CGGTCAAAGGAGGTAG-3′	5′-GGAGCCATAGTCCAATA-3′	60
ATP6v0d2	5′-AGCAAAGAAGACAGGGAG-3′	5′-CAGCGTCAAACAAAGG-3′	60
mitf	5′-TCGTGTGGCAGGATGTGTAT-3′	5′-ACCTGGTGTCAGTCTCAGAGG-3′	62
NFATc1	5′-GAGGAGTTGGCTCAGTG-3′	5′-TAGCGTTCCGTTCGTT-3′	61
OSCAR	5′-GGTCCTCATCTGCTTG-3′	5′-TATCTGGTGGAGTCTGG-3′	62
Irf8	5′-AGACGAGGTTACGCTGTGC-3′	5′-TCGGGGACAATTCGGTAAACT-3′	62
FoxO1	5′-TCGTCATAATCTGTCCCTACACA-3′	5′-CGGCTTCGGCTCTTAGCAAA-3′	61
Nrf2	5′-GGCGTTAGAAAGCATCCTTCC-3′	5′-GCAGAGGGCACACTCAAAGT-3′	61
